# Simultaneous Scanning Electrochemical Microscopy and UV–Vis Absorption Spectroelectrochemistry

**DOI:** 10.1021/acs.analchem.2c05468

**Published:** 2023-06-23

**Authors:** Juan Victor Perales-Rondon, Sheila Hernandez, Ana C. Gonzalez-Baro, Aranzazu Heras, Alvaro Colina

**Affiliations:** † Department of Chemistry, Universidad de Burgos, Pza. Misael Bañuelos s/n, Burgos E-09001, Spain; ‡ CEQUINOR (CONICET, UNLP), Bvd. 120 No 1469, La Plata B1900AVV, Argentina

## Abstract

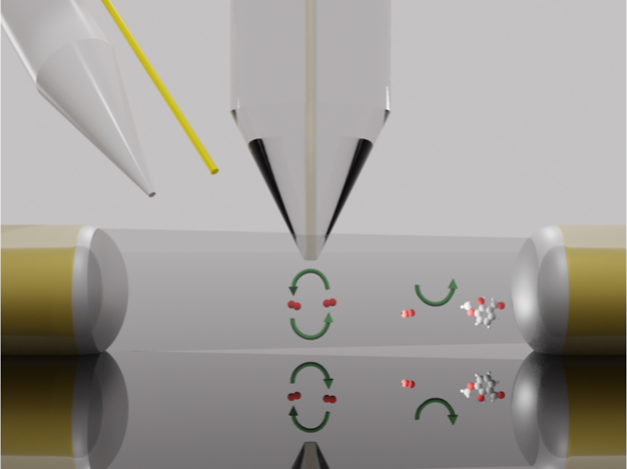

The combination of instrumental techniques allows obtaining precise and reliable information about the reactions taking place at the electrode/solution interface. Although UV–Vis absorption spectroelectrochemistry (UV–Vis SEC) provides a molecular insight about the species involved in the electrode process, obtaining information about the redox state of the products generated in this process is not always accessible by this technique. In this sense, scanning electrochemical microscopy (SECM) has a clear advantage, since it provides additional information on the oxidation state of the intermediates/products. Therefore, the combination of these two techniques facilitates obtaining a more complete picture of the electrochemical reaction studied from two different points of view, but under exactly the same experimental conditions. In this work, the combination of UV–Vis SEC in parallel configuration and SECM is carried out for the first time. This new technique allows distinguishing between those species that are electrochemically active and, at the same time, exhibit changes in the UV–Vis absorption spectra during the electrochemical reaction. The new experimental setup is first validated using ferrocenemethanol as a standard probe, concomitantly obtaining spectroscopic and electrochemical information that accurately describes the oxidation process. Finally, the strength of this combined technique is demonstrated by studying the antioxidant activity of *o*-vanillin (*o*-HVa) in the presence of electrogenerated superoxide. The information extracted from the new UV–Vis SEC/SECM technique makes it possible to identify, beyond any doubt, not only the origin of the electrochemical signals recorded in the SECM tip but also to evaluate the antioxidant effect of *o*-HVa at different concentrations.

## Introduction

The study of electrochemical systems is complex, with many multifactorial parameters governing electron transfer processes at the interface.^1,2^ In this sense, the use of coupled or combined techniques is paramount to understand the reaction mechanisms taking place at the electrode surface.^3,4^ Over the years, spectroelectrochemistry (SEC) has shown a great potential in providing a deep insight into the electrochemical process, and a number of coupled spectroscopic techniques have been developed accordingly.^5−7^ One of them, with many applications in the analytical field, is ultraviolet and visible absorption spectroelectrochemistry (UV–Vis SEC).^8^ This technique provides complementary qualitative and quantitative information about the compounds participating in the electrode reaction.^7,9,10^ In this case, the lack of molecular information of electrochemistry is complemented by the intrinsic molecular character of the spectra recorded during the electrochemical experiment. Very nice examples of application of UV–Vis SEC in the study of a myriad of chemical systems can be found in the literature.^11−13^ However, although most of the molecules present a recognizable UV–Vis spectrum, the technique is limited by the absorption properties of the molecule under study, as well as by the molar absorptivity of such a molecule. To overcome this drawback, the combination of UV–Vis SEC with other complementary techniques such as Raman spectroscopy,^14,15^ among others, is being promoted.

Among the different techniques employed to get insights on the interfacial processes, scanning electrochemical microscopy (SECM) has gained significant recognition over the years.^16^ SECM is able to provide key information about electrochemical processes, owing to the possibility of probing or interrogating the surface with a sensitive ultra-microelectrode (UME) which can be placed as close as possible to the electrode surface.^17−19^ This technique has been used to study a number of chemical systems, from electrochemical deposition to chemical compounds reacting at the electrode.^20−24^ Yet, it is a pure electrochemical technique that provides mainly kinetic information about the reactions taking place on the electrode surface. Additionally, the use of mathematical models to unravel the processes related to the chemical systems involved in the electrochemical process is needed.

Nice examples of combination of SECM with spectroscopic techniques can be found in the literature to obtain relevant operando information about what is happening at the interface. For example, Rodríguez-López et al. have explored the combination of SECM with Raman to study electrochemically active colloids,^25^ or the reactivity of graphene sheets,^26^ demonstrating a successful assembly of both techniques in a single experimental setup. Kranz et al. have tried the *in-situ* infrared attenuated total reflection spectroscopy to monitor microstructured polymer depositions induced by using SECM in the feedback mode.^27^ The combination of fluorescence spectroscopy with SECM has also been addressed by Börsch et al.^28^ Additionally, different combinations of SECM with surface plasmon resonance,^29^ mass spectrometry, or X-ray-based methods have also been reported.^30^

Although different and varied spectroscopic techniques have been explored, to the best of our knowledge, no attempts have been carried out to combine SECM and UV–Vis SEC to interrogate the same portion of solution. Few years ago, performing experiments using these two techniques was challenging owing to the difficulties in controlling the distance between a substrate and the SECM tip and to interrogate the diffusion layer with a light beam in UV–Vis SEC simultaneously. Nowadays, the technological advances in the two techniques have evolved significantly and their combination in a single experiment can be performed much more easily. Moreover, commercial instrumentation is available for the two techniques, facilitating their use.

In the present work, the combination of UV–Vis absorption SEC and SECM techniques is proposed. For this purpose, both UV–Vis SEC in a parallel configuration and SECM will be implemented. In the UV–Vis SEC in a parallel arrangement, the light beam interrogates the solution adjacent to the electrode by passing the light beam grazing the electrode. This arrangement allows us to obtain the spectra of all species that are located in the diffusion layer being able to relate the electrochemical response with the appearance and/or consumption of species in the diffusion layer.^31^ There are different working modes to assemble SECM with SEC. Here, we propose to work in the well-known substrate generation/tip collection (SG-TC) mode during a voltabsorptometric experiment. As an advantage, the light beam passing through the fibers enables us to know the exact position of the tip, being possible to perform an optical approach curve to the electrode, which significantly simplifies the experimental steps prior to performing the SEC assay, while avoiding the use of additional electrochemical reactions that could affect the composition of the electrode/solution interface before performing the experiment.

In order to demonstrate the good performance of the scanning spectroelectrochemistry microscopy (SSECM) setup, ferrocenemethanol (FcMeOH) has been used as a model molecule owing to its typical use as an electrochemical meditator for SECM^32^ and for being a very well-known redox couple used in UV–Vis SEC.^33^ Subsequently, as a proof of concept, the study of the antioxidant capacity of *o*-vanillin (*o*-HVa) has been carried out to illustrate the usefulness of this new combined technique. Finally, it is worth noting that the antioxidant capacity of this molecule has been studied before using both, near-normal and bidimensional UV–Vis SEC.^34,35^ However, herein, we use UV–Vis SEC in a parallel configuration, demonstrating that depending on the experimental design, the same and/or complementary information can be extracted about the same chemical system.

## Experimental Section

### Reagents and Materials

Ferrocenemethanol (FcMeOH, 97%, Sigma-Aldrich), dimethyl sulfoxide (DMSO, 99.8% for HPLC, Merk), tetrabutylammonium hexafluorophosphate (TBAPF_6_, 98%, Merk), and *o*-vanillin (*o*-HVA, Merk) were used as received without further purification. For safety considerations, especially when working with DMSO due to skin absorption at prolonged exposure, all handling and processing were performed carefully during all experiments. Aqueous solutions were freshly prepared, or stored at 4 °C, using ultrapure water (18.2 MΩ cm resistivity at 25 °C, Milli-Q Direct 8, Millipore).

### Instrumentation

All experiments were performed at room temperature. Spectroscopy measurements were performed using a customized UV–Vis SPELEC instrument (Metrohm-DropSens). A deuterium lamp (DH-2000BAL, Ocean Optics) was used as a light source. Bare optical fibers (100 μm in diameter, Ocean Optics) were used to guide the light beam from the lamp to the detector. A CHI 900B potentiostat (CH-Instruments) was employed for SECM measurements. A commercial 10 μm diameter Pt ultramicroelectrode (Pt UME, CH-Instruments) was used as tip while a glassy carbon (GC) foil (Goodfellow) was used as substrate. The counter and pseudo-reference electrodes were a gold wire and a silver wire, respectively. Prior to the SECM measurements, the Pt UME was polished and cleaned electrochemically by performing several voltammetric cycles in 0.5 M H_2_SO_4_ solution. An integration time of 100 ms was used in the optical experiments.

### SSECM Cell

**Figure 1:**
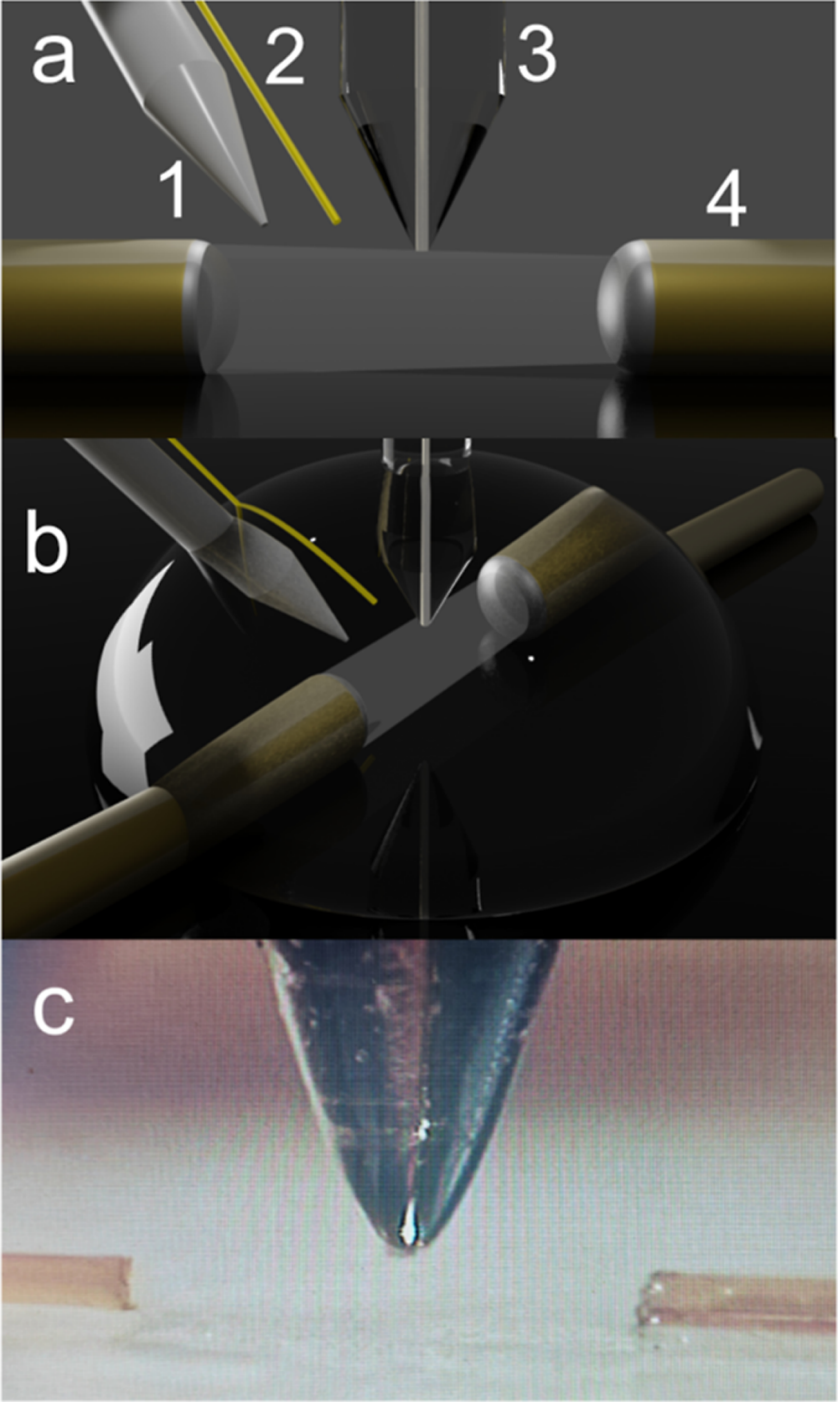
(a) Schematic of the SSECM cell, where the positions of the pseudo-reference electrode (1), the gold counter electrode (2), and the Pt UME (3) are shown. The light beam passing through the two optical fibers placed on top of the GC substrate (4) is also represented. (b) Schematic of the tip placed between the two optical fibers using a GC foil as the substrate from another point of view and after placing the drop of solution. (c) Real image of the system used in the SSECM setup.

Figure 1 shows a schematic of the electrochemical cell used to perform SSECM measurements. Two 100 μm optical fibers were attached to a GC foil using nail polish. The distance between the two optical fibers was 0.45 mm. A silver wire and a gold wire were placed at some millimeters from the optical fibers to avoid any interference of the products generated on the counter electrode. The tip was placed in the optical pathway, between the two optical fibers. SECM micro positioners were used to place the tip between the two optical fibers. The intensity of light recorded in the spectrometer helps to place the tip in the correct position in an easy way. Figure 1a,b show different views of the SSECM cell, whereas Figure 1c shows a real image of SECM tip placed between the two optical fibers using GC as substrate. Figure S1 in the Supporting Information file shows a photography of the SSECM setup from another perspective.

## Results and Discussion

### Electrochemical and Optical Approach Curve

An important advantage of combining UV–Vis SEC and SECM is the possibility of easily estimating the distance between the tip and the substrate, GC in this case. Once the tip is placed between the two fibers, it must be retracted to avoid any interaction with the light beam. After that, it can be approached to the substrate while the light intensity is monitored in the spectrometer, so an approach curve can be obtained. To achieve that, light intensity at a selected wavelength is plotted with respect to the distance traveled by the tip, obtaining an optical approach curve, Figure S2a.

The point where the light beam decreases coincides with the height of the optical fiber, which in this case is 130 μm, due to the cladding of the optical fibers. As can be seen in the optical approach curve (Figure S2a), in step (1), the light remains stable during the initial stage of the tip advancing into the fibers (Figure S2b).

In step (2), the light intensity starts to decrease when the tip is aligned with the beginning of the cross section of the optical fibers (Figure S2c). Finally, in step (3), the light intensity drops drastically when the tip is approached to the substrate, since the electrode is hindering the light that reaches the collecting optical fiber (Figure S2d).

The accuracy of the optical approach curve was calculated by comparison with a classical approach curve using the SECM feedback mode. Figure S3 shows the comparison of these two approach curves using a Pt UME (10 μm) with an RG = 5. As can be confirmed from the figure, when the electrode is close to the substrate, the optical approach curve reaches a minimum that coincides with the end of the fiber section, 100 μm. At this point, the electrode is at 30 μm with respect to the substrate, which is calculated by the feedback mode approach curve. This result emphasizes the reliability and usefulness of an optical approach curve for approaching the tip to the substrate, especially in cases where the use of an electrochemical reaction has to be avoided.

Once the tip is placed at a desired distance from the substrate, the electrode process taking place on the electrode can be interrogated using both the UME and the optical fibers. For this purpose, in this setup, the SECM tip should be placed at 130 μm from the substrate surface.

### Validation of the SSECM Setup Using Ferrocenemethanol

FcMeOH is a very well-known molecule that is usually employed as mediator to validate the SECM setup as well as reference probe in UV–Vis SEC experiments.^32,33^ A substrate generation-tip collection (SG-TC) experiment was carried out to demonstrate the appropriate performance of the experimental setup. The validation assay consisted in performing a cyclic voltammetry (CV) using a GC substrate as the main WE. A 10 μm Pt tip was used to collect the products generated on the substrate during the CV (UME WE), by applying −0.20 V. It is worth noting that the tip was placed at 130 μm from the GC surface by performing the corresponding optical approach curve, as was described above.

**Figure 2:**
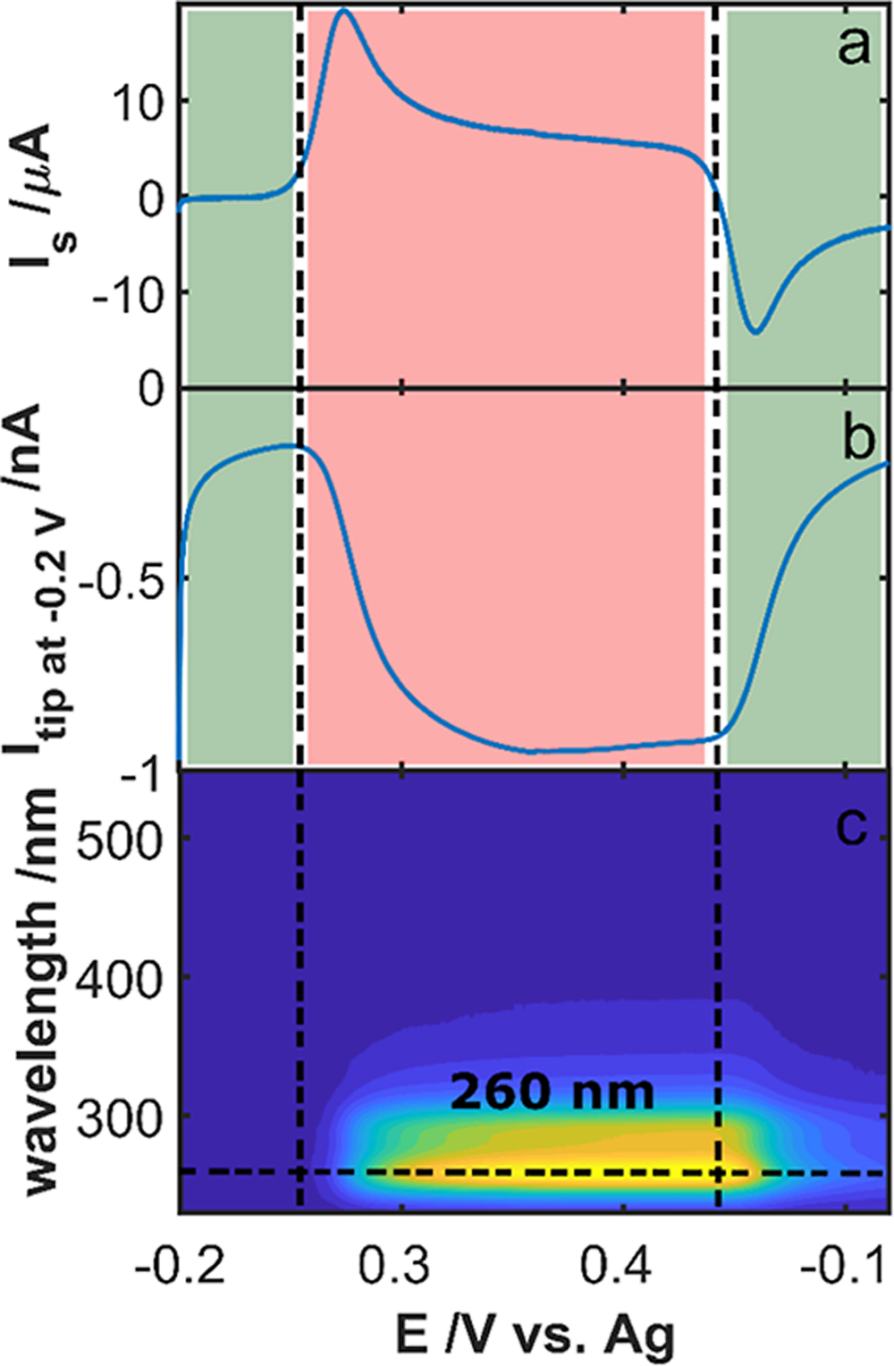
(a) CV for the oxidation of FcMeOH on a GC electrode. (b) Current registered for the tip polarized at −0.20 V. (c) Contour plot for the evolution of the spectra in the diffusion layer with the applied potential. The experiment was carried out in a solution containing 5 × 10^–4^ M FcMeOH and 0.1 M KCl. CV experiment between −0.20 V and +0.60 V at 0.01 V s^–1^. Tip potential: −0.20 V.

Figure 2 shows the three responses obtained during the SSECM experiment. Figure 2a displays the CV for the oxidation of FcMeOH at the substrate. As can be distinguished, an oxidation current starts to appear at a potential +0.04 V that is assigned to the conversion of Fe(II) to Fe(III) in the FcMeOH molecule. This oxidation process extends up to the potential of +0.23 V in the reverse scan, where starts to appear a reduction current, related to the reverse process, reduction of ferroceniummethanol (FcMeOH^+^) to FcMeOH. This can be confirmed by the tip current registered in Figure 2b, where a negative current appears concomitantly with the FcMeOH oxidation. This cathodic current decreased during the reduction of FcMeOH^+^ on the substrate. It is noteworthy that a negative background in the current of the tip is always present. This constant cathodic current comes from the oxygen reduction reaction taking place on the tip surface, particularly relevant for Pt UMEs. Nevertheless, this cathodic background does not affect the collection of products in the tip for this particular experiment. UV–Vis absorption spectra measurements were simultaneously obtained during the CV, obtaining the evolution of UV–Vis spectra, shown as contour plot in Figure 2c. A UV absorption band centered at 260 nm, corresponding to the generation of FcMeOH^+^, is observed when the solution adjacent to the electrode is interrogated by the optical fibers. This signal only appears in the potential region delimited by the oxidation of FcMeOH (pink region). The individual response of each measurement, the CV obtained in the substrate, the current obtained in the tip, the evolution of the absorbance as a function of the applied potential (voltabsorptogram) at 260 nm, and the spectra at the peak potential are plotted in Figure S4. The good agreement between the different responses demonstrates the correct performance of the experimental setup that surely can be used to study more complex electrochemical systems.

Having proven the usefulness of the experimental setup to carry out a SG-TC SECM mode, an additional evaluation for a tip generation-substrate collection (TG-SC) mode was also carried out. A similar experiment, where FcMeOH is oxidized in the tip and collected in the substrate, is shown in Figure S5. As can be clearly seen in the Figure S5, when the FcMeOH is oxidized to FcMeOH^+^, there is not current detection in the substrate. Similar experiments were performed at a lower distance tip-substrate (50 μm), but still no significant current was detected in the substrate. On the contrary, since the tip is in the region defined by the cross section of the fibres, the detection of FcMeOH^+^ by the appearance of an absorbance signal at 260 nm when FcMeOH is oxidized in the tip is registered, demonstrating that this coupled technique could be really interesting not only in those cases where a product is not an electroactive species (but has a UV–Vis response), but also in those cases where the amount of product generated in the tip is really small to be clearly detected in the substrate when working in the TG-SC mode.

### Study of *o*-HVa as an Antioxidant

Antioxidant capacity is a very interesting property to categorize natural compounds for their ability to reduce the oxidative effect in food and human health.^36^ In particular, antioxidant agents fight against the well-known reactive oxygen species (ROS) such as superoxide (O_2_^•–^) radical ion. Under normal concentrations, ROS can mediate key cellular processes. However, accumulation and/or overproduction of them is related to the onset and progression of several neurological and cardiovascular diseases, aging, or obesity.^37^ Compounds containing phenolic groups are ideal candidates that have proven to have a high antioxidant capacity.

The standard protocol to assess antioxidant capacity of a molecule consists of evaluating the reaction activity with superoxide ion in a non-aqueous solvent. Although electrochemical methods are the gold standard technique to study this process,^38^ the use of UV–Vis-SSECM holds great promise for providing more accurate information on antioxidant behavior.

**Figure 3:**
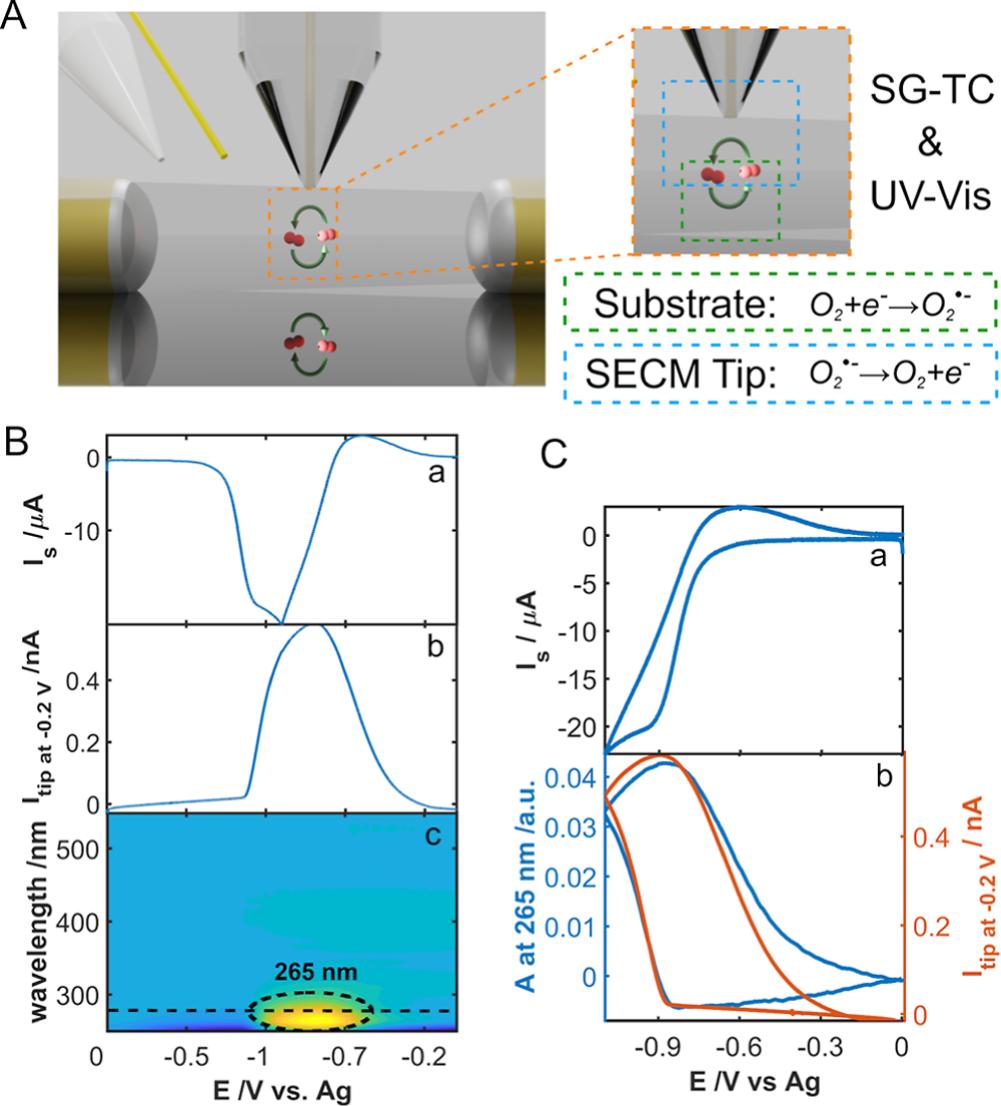
(A) Schematic of the superoxide formation and detection using the UV–Vis-SSECM setup (left side). On the right side is shown a close-up view with the corresponding electrochemical processes taking place in the substrate and the SSECM tip. (B) Current registered in the substrate for the O_2_ reduction reaction to form superoxide (a). Current registered for the Pt tip polarized at −0.20 V (b). Contour plot for the evolution of the absorption spectra in the diffusion layer during CV (c). (C) CV curve for the substrate (a). Current for the tip at −0.20 V (orange curve) compared with the voltabsorptogram of the system at 265 nm (blue curve) (b). The experiment was carried out in an air-saturated DMSO solution containing 0.1 M TBAPF_6_ as the supporting electrolyte. CV experiment between 0 and −1.10 V at 0.01 V s^–1^. Tip potential: −0.20 V.

*o*-Vanillin (*o*-HVa) has been proposed as a model in the study of antioxidant capacity. In a regular procedure, superoxide ion is generated during the reduction of oxygen in an aprotic solvent such as DMSO. The presence of an anhydrous aprotic solvent is mandatory (i) to avoid any contribution from hydrogen evolution in this potential region and (ii) to increase stability of superoxide in such electrolytic conditions. Following a previously described procedure,^35^ a 10 μm Pt tip was used to collect the superoxide ion generated on the substrate during the CV in an air-saturated DMSO solution containing 0.1 M TBAPF_6_ as supporting electrolyte. Figure 3A depicts the schematic of the processes taking place during the SSECM experiment. Particularly, the two main processes are represented: (a) on the one hand, the formation of superoxide as a result of the oxygen reduction reaction on the substrate (green dashed box) and, (b) on the other hand, the detection of the superoxide in the Pt UME (blue dashed box). Figure 3B shows the three main responses obtained in a UV–Vis-SSECM experiment during a CV.

In Figure 3Ba, the superoxide generation at the substrate can be distinguished, which is simultaneously detected both with the tip and with the optical fibers, as shown in Figure 3Bb,c, respectively. As can be observed in Figure 3Ba, the cathodic current is related to the production of superoxide in these electrolytic conditions. The current of the tip confirms the opposite process, which is the oxidation of superoxide at −0.20 V, as has been previously demonstrated in the literature.^35^ Finally, in the region of superoxide production, a UV absorption band centered at 265 nm can be observed, assigned to the formation of superoxide. A further representation of the CV and the rationalized signals obtained both, at the tip and with the optical fibers, can be found in Figure 3C. As can be observed, the current registered at the tip (orange curve, Figure 3Cb) shows a similar shape than the voltabsorptogram at 265 nm (blue curve, Figure 3Cb), which corresponds to the evolution of the absorption band of superoxide with the applied potential. This is a double confirmation of the generation of superoxide in such electrolytic conditions. It is worth noting that the monitoring of this system with UV–Vis SEC allows us to confirm the nature of the electroactive species formed in the electrochemical experiment. The latter represents an advantage to distinguish between products and intermediates during an interfacial process that is not easy to obtain by using only the SECM setup.

**Figure 4:**
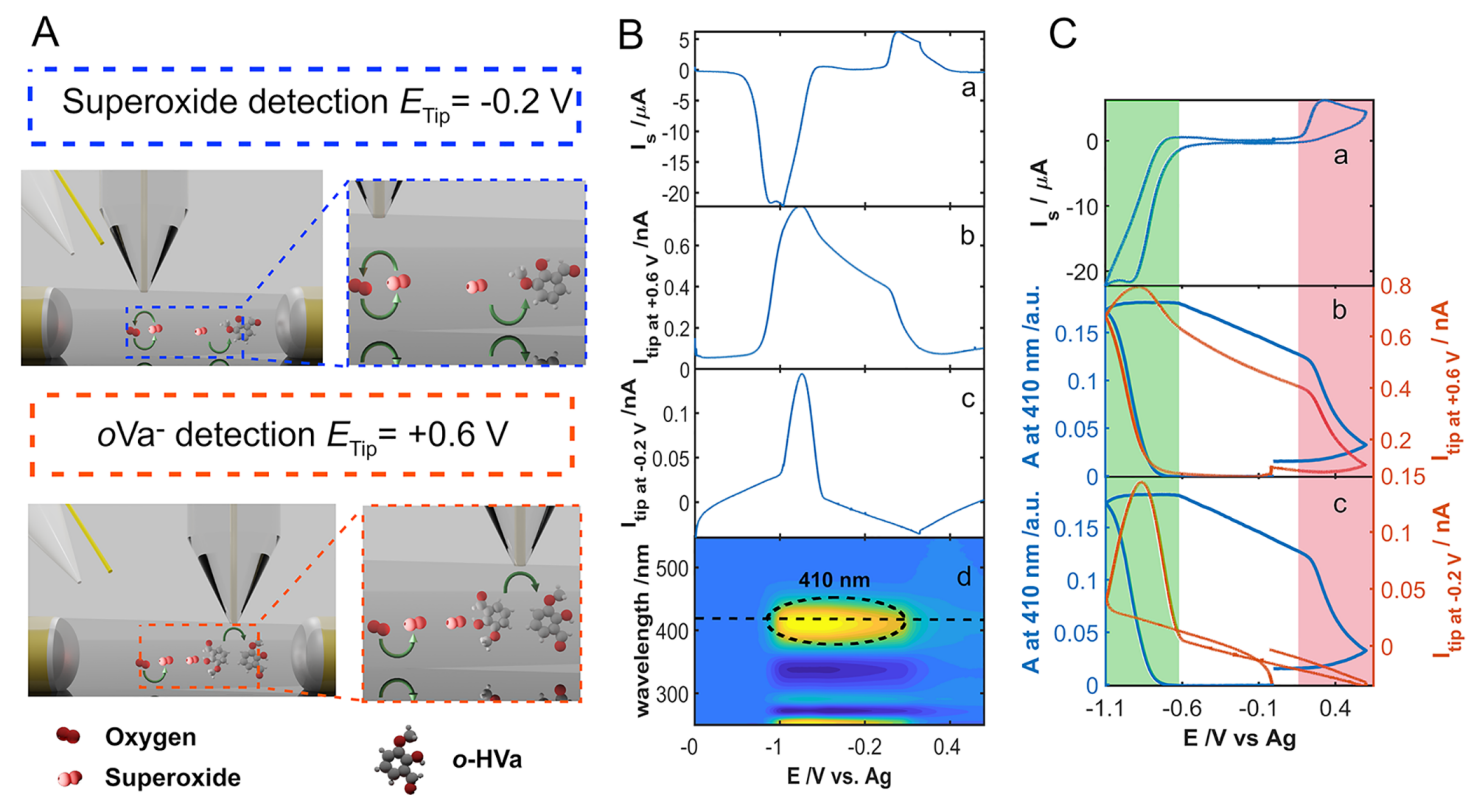
(A) Schematic of the superoxide formation and detection using the SSECM setup. Top: schematic of the system when detecting superoxide in the tip. Bottom: schematic of the system when detecting *o*-Va^–^ in the tip. (B) Current registered in the substrate for the O_2_ reduction reaction to form superoxide (a). Current registered for the Pt tip polarized at +0.60 V (b) and −0.20 V (c). Contour plot for the evolution of the spectra in the diffusion layer with the applied potential (d). (C) CV curve for the substrate (a). Current for the tip at +0.60 V (orange curve) compared with the voltabsorptogram of the system at 410 nm (blue curve) (b). Current for the tip at −0.20 V (orange curve) compared with the voltabsorptogram of the system at 410 nm (blue curve) (c). The experiment was carried out in an air-saturated DMSO solution containing 0.1 M TBAPF_6_ as the supporting electrolyte and 5 × 10^–4^ M *o*-HVa. CV experiment between +0.45 V and −1.10 V at 0.01 V s^–1^. Tip potential: −0.20 V.

Once demonstrated the successful study of superoxide formation and detection, a similar experiment in presence of *o*-HVa was performed, to study its antioxidant capacity. According to the literature, the antioxidant mechanism follows a process that involves the superoxide formation and the subsequent chemical reaction with the antioxidant to yield the corresponding anionic species, in our case *o*-Va^–^, as has been previously described.^35,38^ This means that we can either follow the production of O_2_^•–^ or the generation of *o*-Va^–^ at the solution adjacent to the electrode. Figure 4 shows the main electrochemical and spectroscopic responses of superoxide generation in presence of *o*-HVa by using UV–Vis-SSECM technique. Figure 4A depicts a schematic of the whole process taking place during the UV–Vis-SSECM experiment. In the top side is shown the generation and simultaneous detection of superoxide at the tip and sampled by the UV–Vis optical fibers, whereas in the bottom part, the formation of *o*-Va^–^ and the corresponding oxidation at the tip is presented. As can be observed in Figure 4Ba, there are two main well-defined regions, the cathodic one, related to the generation of superoxide radical, and the anodic one, related to the oxidation of the anion *o*-Va^–^, as has been previously reported in the literature.^35^ Besides, Figure 4Bb depicts the current of the tip at +0.60 V in which the *o*-Va^–^ oxidation is achieved, while the oxidation of superoxide is detected at the tip polarized at −0.20 V (Figure 4Bc), as in the previous experiment. Finally, the UV–Vis spectra for the evolution of the system during the electrochemical experiment is shown in Figure 4Bd, where a maximum absorbance at 410 nm is shown, corresponding to the formation of the *o*-Va^–^ species. To better understand the whole experiment, the corresponding CV of the substrate is shown in Figure 4Ca. On the other hand, Figure 4Cb shows a comparison of the voltabsorptogram at 410 nm and the tip current at +0.60 V. As can be seen, both signals follow the same trend, indicating that they correspond to the same species. According to the literature, the *o*-Va^–^ anion resulting from the reaction with the superoxide can be oxidized at +0.60 V. Besides, the UV–Vis absorption spectrum for *o*-Va^–^ presents a strong band at 410 nm. This information confirms that both signals correspond to the formation of *o*-Va^–^ anion after the chemical reaction with the superoxide. Another demonstration of the origin of these two signals can be found in the comparison of the CV and the derivative of the voltabsorptogram at 410 nm (Figure S6). As can be observed from the figure, the inverse of the derivative signal reproduces reasonably well the shape of the CV, which reveals that the voltametric currents are directly related with the production of *o*-Va^–^.

Interestingly, when evaluating the signals presented in Figure 4Cb, there is a change of the trend around −1.10 and −0.60 V (green zone), where a slight increase in the tip current is recorded. This slight increment can be related to the excess of superoxide formed at this potential region. In fact, when evaluating the current of the tip at −0.20 V (Figure 4Cc), the presence of superoxide in this region is clearly shown, with a net increment of ca. 0.1 nA (Figure S7). An additional demonstration of that is found in the difference between the current of the tip (*C*_Tip_) at +0.60 V minus the *C*_Tip_ at −0.20 V (Figure S8). After this, the current of the tip matches well the behavior of the voltabsorptogram at 410 nm, which demonstrates the origin of this discrepancy. The latter emphasizes the importance of using SEC signal, since it helps to clearly differentiate between those species that can be oxidized or reduced at a certain potential. Indeed, the information that cannot be assessed by only using electrochemical techniques can potentially be resolved using SEC tools, as long as the products and/or reagents give a recognizable UV–Vis response.

When performing a SECM experiment, considering the redox competition between the tip and the substrate is highly relevant, especially to understand and characterize the influence of one to each other in their redox response. In fact, upon registering the current at the tip, two different regions where redox competition takes place can be clearly found. On the one hand, the abrupt fall in the current of the tip at −0.20 V (green region, Figure 4C) can be ascribed to the oxidation of some remaining superoxide in the substrate. On the other hand, the second fall in the current of the tip at +0.60 V (pink region), which is due to the oxidation of *o*-Va^–^ in the substrate, can also be followed by the decrease in the absorbance at 410 nm (Figure 4Cb,c). These two examples reveal the strong influence of the substrate on the response of the tip, when working in SECM, and highlight the usefulness of the combination with UV–Vis SEC to deconvolve and better understand the whole interfacial phenomenon.

**Figure 5:**
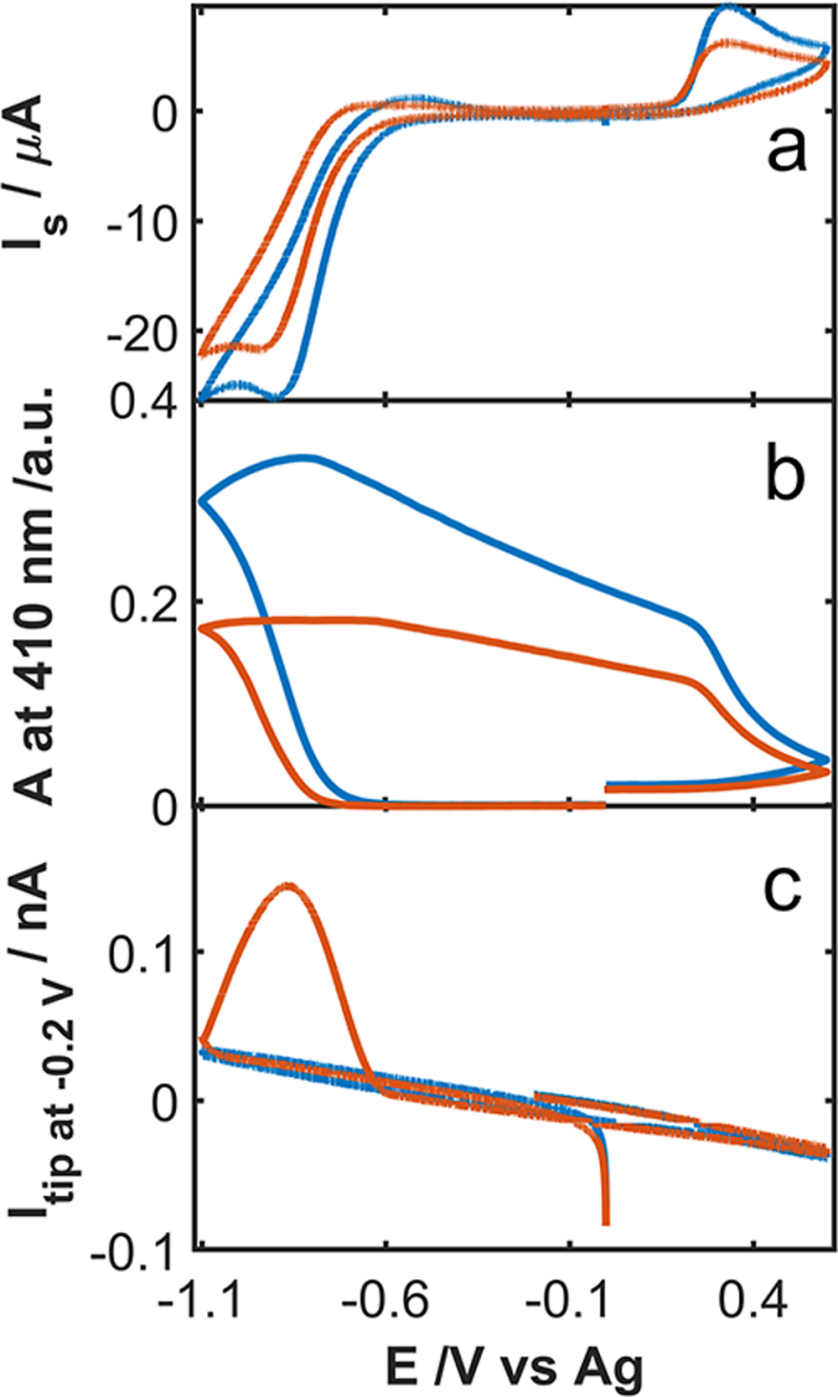
Comparison of the antioxidant capacity of *o*-HVa at two different concentrations: 5 × 10^–4^ M (orange curve) and 1 × 10^–3^ M (blue curve). (A) CV curve for the substrate. (B) Voltabsorptogram of the system at 410 nm. (C) Current for the tip at −0.20 V. The experiment was carried out in an air-saturated DMSO solution containing 0.1 M TBAPF_6_ as the supporting electrolyte. CV experiment between +0.45 and −1.10 V at 0.01 V s^–1^. Tip potential: −0.20 V.

This brand-new technique can also be used to assess the influence of *o*-HVa concentration on the antioxidant capacity. Figure 5 shows a comparison of the response obtained during a UV–Vis-SSECM experiment at two different concentrations of *o*-HVa. From the current registered at the substrate, it can be inferred that the experiments are similar in terms of superoxide formation (Figure 5A). Furthermore, the evolution of the spectra at 410 nm for two different concentrations of *o*-HVa (Figure 5B) shows that at 1 × 10^–3^ M the amount of *o*-Va^–^ produced is higher than that at 5 × 10^–4^ M. Considering that *o*-Va^–^ only comes from the chemical reaction with superoxide, and that the amount of superoxide formed is similar, it is inferred that the *o*-HVa content has been shortened in the experiment with 5 × 10^–4^ M. This can be further confirmed in Figure 5C, where the current of the tip at −0.20 V (orange curve) shows a remaining superoxide concentration that is detected at lower potentials (form ca. −1.10 to −0.60 V). This means that the concentration of *o*-HVa is not enough to suppress all superoxide electrogenerated during the CV. This is consistent with the observation of the voltabsorptogram at 410 nm, where a flat signal is observed at this potential region, which indicates that when the production of superoxide is increasing, even so, no increase in the optical signal for *o*-Va^–^ is registered. However, when increasing the concentration of the antioxidant, no signal for the tip at −0.20 V is achieved (blue curve, Figure 5c). Therefore, a concomitant increase in the optical signal at this potential region is obtained, confirming, in this case, that the concentration of the antioxidant is enough to suppress all superoxide formed during the electrochemical test. This is particularly useful in the quantitative evaluation of the antioxidant capacity.

## Conclusions

In conclusion, a new experimental setup consisting of the combination of UV–Vis SEC in parallel configuration with the SECM technique has been presented. The new setup has been properly validated using FcMeOH as a probe molecule in a regular SEC experiment. Further, the potential of the UV–Vis-SSECM has been demonstrated with the study of the antioxidant capacity of *o*-HVa in different concentrations. The use of this technique allows not only the identification of different species involved in the antioxidant mechanism but also the correlation between the electroactive species with those that additionally present changes in the spectroscopic response. Simultaneous UV–Vis absorption SEC and SECM allows users to obtain a double and independent information of exactly the same system under the same conditions. Sometimes, for a studied process, data obtained in sequential experiments can have non-correlated or contradictory results because of any anomalous evolution of the system. However, this brand-new technique would provide different responses in the two signals for each experiment. Therefore, the combination presented in this work is just one technique but much more powerful. This is the first time that such useful combination has been carried out, and we anticipate that this technique will be very useful to assess the mechanism of complex interfacial phenomena.

## Supporting Information Available

Real pictures of the experimental setup, a scheme of the optical approach curve experiment, UV–Vis spectra, voltabsorptograms, CV curves of the substrate, current of the tip, and derivatives of the voltabsorptograms (PDF)
