# Supplementary material for: Simultaneous Scanning Electrochemical Microscopy and UV–Vis Absorption Spectroelectrochemistry
Source: Anal Chem. 2023 Jun 23;95(28):10532–9. doi: 10.1021/acs.analchem.2c05468 (PMC10357403; doi:10.1021/acs.analchem.2c05468)
Supplement: Supplementary file 1 — ac2c05468_si_001.pdf [file ac2c05468_si_001.pdf]

Supporting Information

Simultaneous Scanning Electrochemical Microscopy and UV−Vis absorption Spectroelectrochemistry

Juan Victor Perales-Rondon*[1], Sheila Hernandez[1], Ana C. Gonzalez-Baro[2], Aranzazu Heras[1], Alvaro Colina*[1]

[1] Department of Chemistry, Universidad de Burgos, Pza. Misael Bañuelos s/n, E-09001 Burgos (Spain)

[2] CEQUINOR (CONICET, UNLP), Bvd. 120 N° 1469, B1900AVV La Plata (Argentina)

Email: acolina@ubu.es; jvperales@ubu.es

Table of content

Images of the cell assembled for UV-Vis-SSECM experiments S-3

Optical approach curve for the UV-Vis-SSECM experiment S-4

Comparison of the optical approach curve with the approach curve obtained by feedback mode of SECM .S-5

Representation of the main signals obtained in the UV-Vis-SSECM experiment for FcMeOH S-6

Representation of the main signals obtained in the UV-Vis-SSECM experiment for FcMeOH in a TG-SC experiment S-7

Comparison of the inverse derivative voltabsorptogram at 410 nm with the CV of the substrate in DMSO and $5 \cdot 10^{-4}$ M *o*-Hva S-8

Representation of the signals for the tip polarized at two different potentials registered during the UV-Vis/SECM experiment for DMSO and $5 \cdot 10^{-4}$ *o*-Hva S-9

Comparison of the voltabsorptogram at 410 nm with the subtraction of the $I_{tip\ at\ +0.60\ V} - I_{tip\ at\ -0.20\ V}$ in the UV-Vis/SECM experiment for DMSO and $5 \cdot 10^{-4}$ *o*-Hva S-10

References S-10

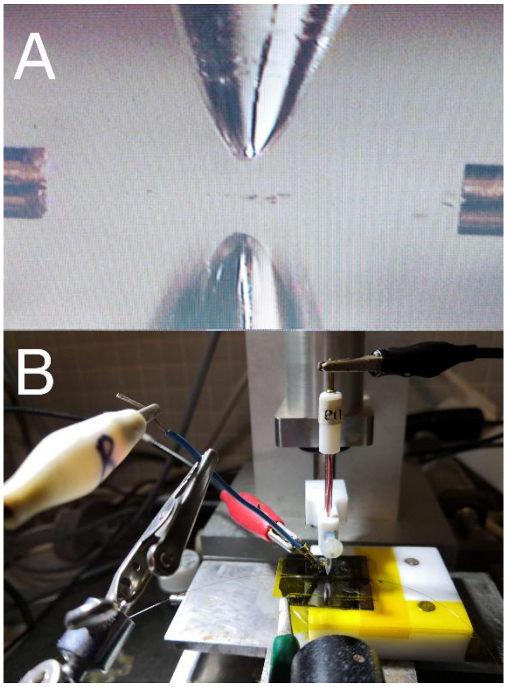

Figure S1. Image of the cell assembled for UV-Vis-SSECM experiments. (a) Close-up view with a magnifying lens. (b) Full view of the assembly developed.

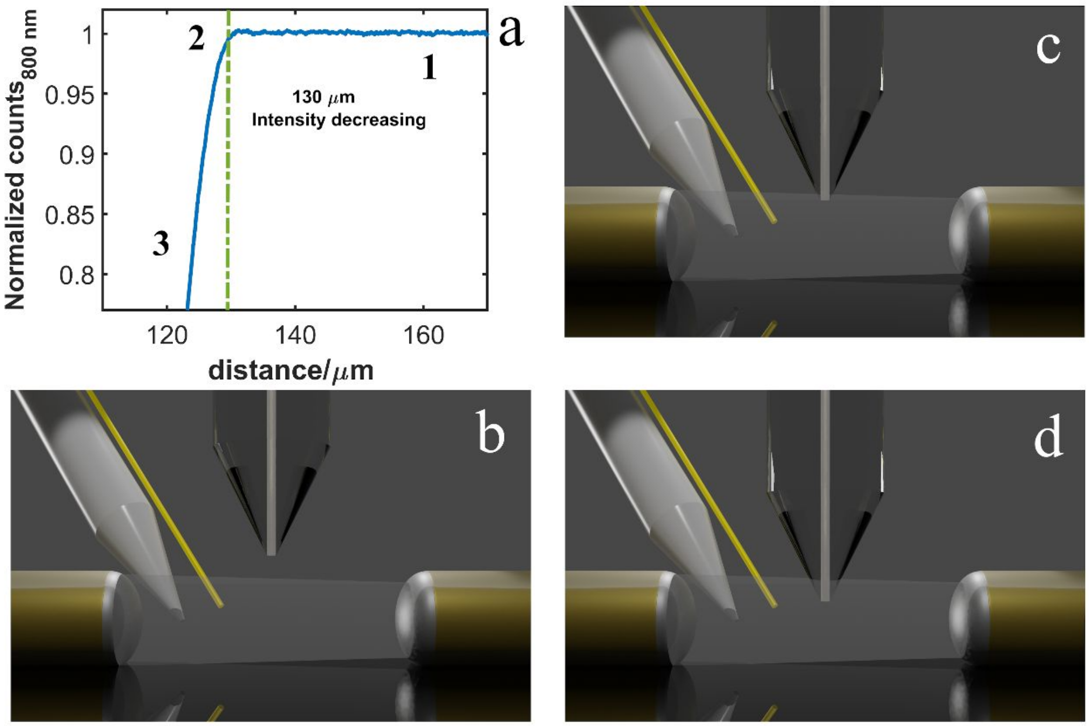

Figure S2. (a) Optical approach curve for the UV-Vis-SSECM experiment. The plot represents the normalized light intensity at a selected wavelength (800 nm) as a function of the distance traveled by the SECM tip. The different stages of the optical approach curve are schematically represented in (b) stage 1, (c) stage 2 and (d) stage 3.

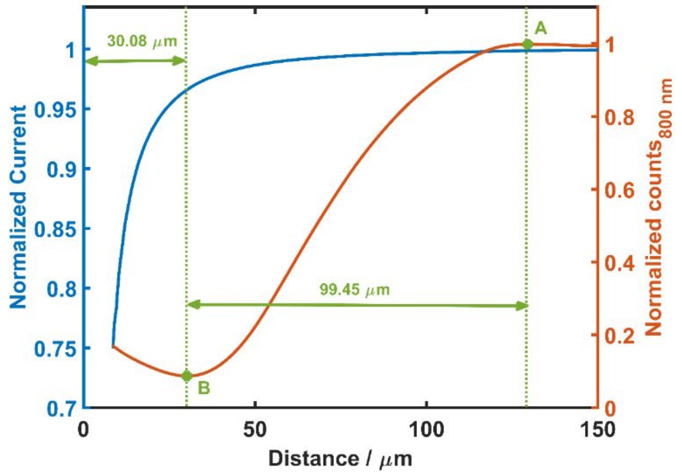

Figure S3. Comparison of the *optical approach curve* (orange, solid line), with the approach curve obtained by feedback mode of SECM (blue, solid line). The experiment for the approach curve was perform by reproducing the experimental setup shown in Figure S1. Electrolytic conditions: $5\cdot10^{-3}$ M FcMeOH and 0.1 M KCl. The experiment was performed with an insulator as a substrate to carry out a negative feedback curve. A 10 μm Pt UME was used as a tip, while a silver wire and gold wire were used as pseudo-reference and counter electrode respectively. The tip was polarized at + 0.60 V vs Ag.

As can be observed in the Figure S3, there is a clear relationship between the two approach curves. In the case of the feedback mode approach curve, the current normalized respect to the initial current is presented. It is important to mention that, after measuring the RG value of the tip (RG = 5), a proper correlation between the theoretical approach curve and the experimental data was demonstrated. An approach curve reducing the initial current to a 75 % of its initial value was performed to be sure that we were at a distance around 1.7 L, which in this case is equivalent to 8.5 μm from the substrate. This calculation allows us to make sure that we travelled through all the light beam section defined by the optical fibres (100 μm). As can be distinguished in the *optical approach curve* (orange, solid line) there are two important points to consider. The first one, point A, is defined as the distance where the tip starts to enter between the optical fibres section, which coincides with the initial diminution of light. The second one, point (B), is the minimum in the *optical approach curve*, which coincides with the point in which the tip has crossed the whole section of the optical fibres. To demonstrate this, a derivative of the light was calculated to clearly found the points A and B. The exact difference of these two points found by the derivative of the spectroscopic signal coincides with the diameter of the fibres, as was initially stated. This finding demonstrates that an *optical approach curve* can be done and could clearly inform about the distance between the tip and the substrate, provided that the fibres are perfectly aligned (parallel) to the substrate and well-characterized.[1]

To further demonstrate the latter, we will compare the value for the cladding of the fibres obtained in an independent work[1] with that obtained in this work (distance at point B), considering that fibres have been aligned following the same experimental protocol and taking into account the differences between operators. Both values are close to 30 μm, which confirms that, upon a correct alignment of the fibres, the exact distance tip-substrate can be estimated/calculated.

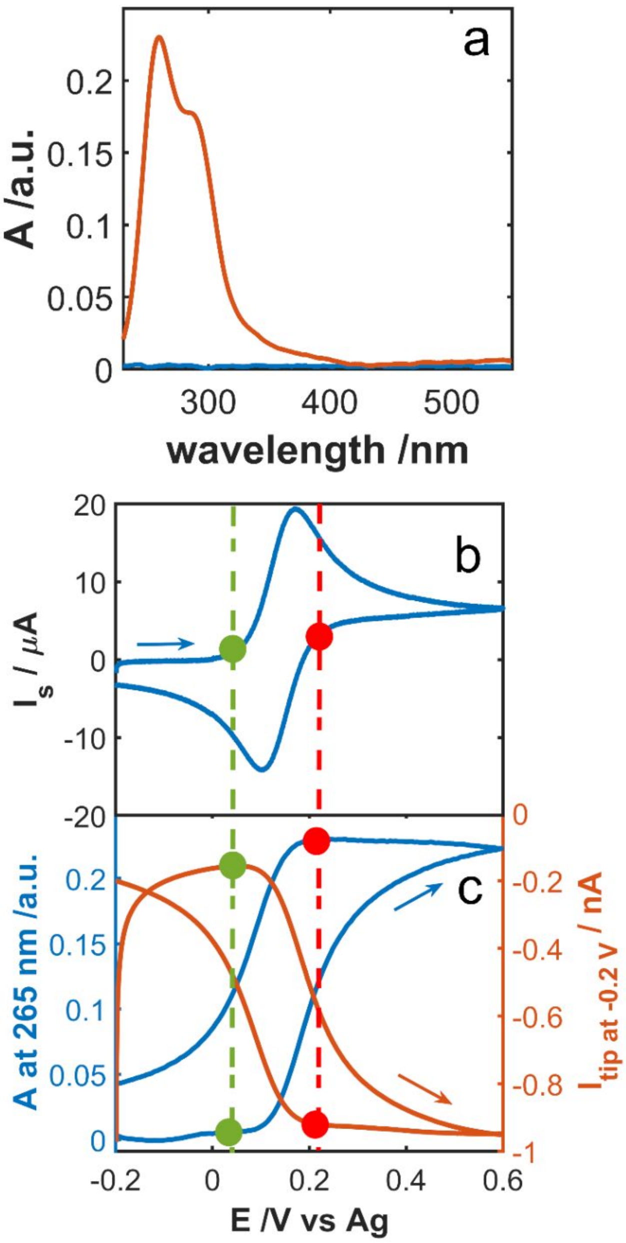

Figure S4. Representation of the main signals obtained in the UV-Vis-SSECM experiment. (a) Spectrum of the $FcMeOH^+$ (orange curve) at the peak potential (+0.23 V), compared with the FcMeOH spectrum at the beginning of the experiment at open circuit potential (taking this spectrum as reference). (b) CV during the oxidation of the FcMeOH at the substrate. (c) Comparison of the current at the tip polarized at -0.20 V with the voltabsorptogram at 260 nm (absorbance of the oxidized product, ferroceniummethanol, $FcMeOH^+$). Dashed green line represents the onset potential for the FcMeOH oxidation. Green circles indicate the potential where the signals related to $FcMeOH^+$ start to appear. Likewise, dashed red line represents the onset potential of the reversed process (reduction of Fe(III) to Fe(II) in the FcMeOH molecule), and red circles indicate the potential at which the signals related to the regeneration of FcMeOH start to appear.

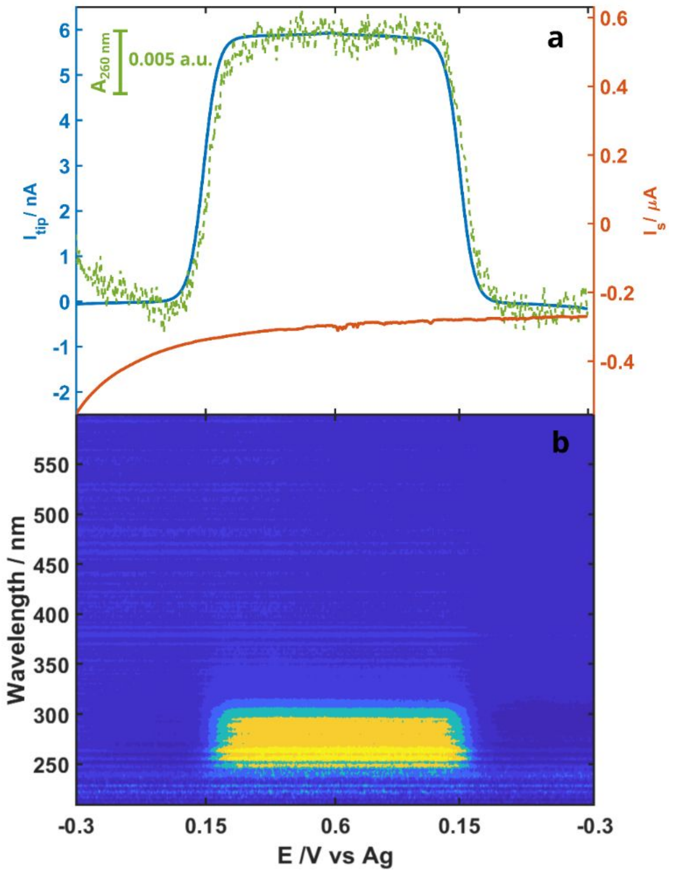

Figure S5. (a) Current of the tip (blue solid line) and evolution of the absorbance at 260 nm (green dashed line), compared with the current at the substrate (orange solid line) obtained in a TG-SC mode with the substrate polarized at -0.20 V vs Ag. (b) Contour plot of the UV-Vis absorption spectra in the optical pathway between the optical fibres recorded during the TG-SG SECM experiment. The experiment was carried out using the experimental setup shown in Figure 2 in the manuscript. Electrolytic conditions: $5 \cdot 10^{-3}$ M FcMeOH and 0.1 M KCl. The experiment was performed using a GC as a substrate with a confined geometric space in contact with the solution. A 10 µm Pt UME was used as a tip, placed at 80 µm from the substrate. A silver wire and a gold wire were used as pseudo-reference and counter electrode, respectively.

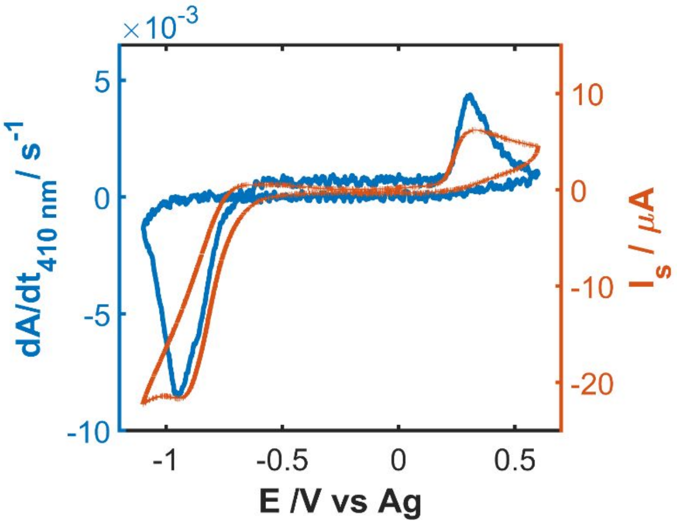

Figure S6. Comparison of the inverse derivative voltabsorptogram at 410 nm (blue curve) with the CV of the substrate (orange curve). Both signals follow the same trend and similar shapes. It is necessary to obtain the inverse of the derivative of the absorbance since, in this case, the spectroscopic signal is inversely proportional to the voltammetric response. The experiment was carried out in an air-saturated DMSO solution containing 0.1 M $TBAPF_6$ as supporting electrolyte and $5x10^{-4}$ M *o*-HVa. CV experiment between +0.45 V and -1.10 V at 0.01 V s^{-1}.

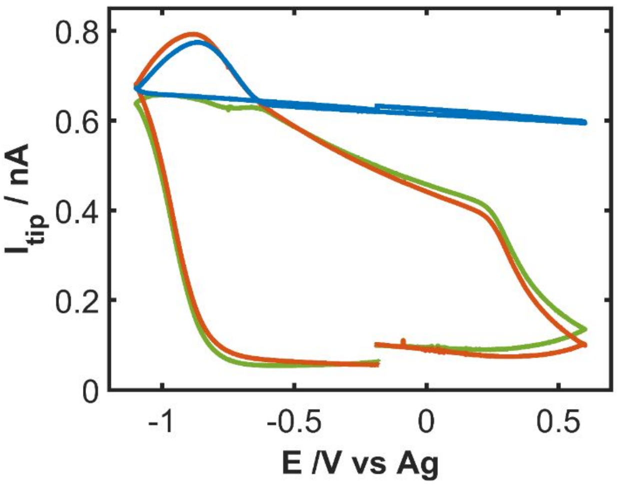

Figure S7. Representation of the signals for the tip polarized at two different potentials registered during the UV-Vis-SSECM experiment. Current of the tip at +0.60 V (orange curve), current of the tip at -0.20 V (blue curve), and subtraction of the $I_{tip\ at\ +0.60\ V} - I_{tip\ at\ -0.20\ V}$ (green curve).

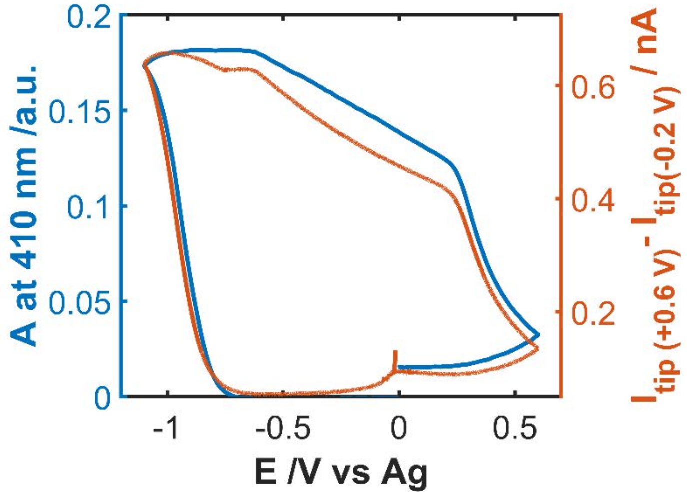

Figure S8. Comparison of the voltabsorptogram at 410 nm (blue curve) with the subtraction of the $I_{tip\ at\ +0.60\ V}$ – $I_{tip\ at\ -0.20\ V}$ (orange curve). Both signals match reasonably well.

References

(1) Romay, L.; González, J.; Molina, Á.; Laborda, E. Investigating Comproportionation in Multielectron Transfers via UV-Visible Spectroelectrochemistry: The Electroreduction of Anthraquinone-2-Sulfonate in Aqueous Media. *Anal. Chem.* **2022**, *94* (35), 12152–12158. https://doi.org/10.1021/acs.analchem.2c02523.
